# Disease Control, Activity and Quality of Life in Chronic Spontaneous Urticaria: A Cross-Sectional Study from Kazakhstan

**DOI:** 10.3390/diagnostics16050672

**Published:** 2026-02-26

**Authors:** Aray Batyrbayeva, Zhanat Ispayeva, Marat Pashimov, Rustem Tuleutayev, Jamilya Kaibullayeva, Madina Baidildayeva, Balnur Sultanova, Arailym Maldybayeva, Kamila Turebekova

**Affiliations:** 1 Republican Allergy Center, JSC Research Institute of Cardiology and Internal Diseases, Almaty 050000, Kazakhstan; aygosh3@gmail.com; 2 Department of Allergology, Asfendiyarov Kazakh National Medical University, Almaty 050000, Kazakhstan; ispayeva.zh@kaznmu.kz; 3 Department of Public Health of Almaty, Almaty 050000, Kazakhstan; pashimovm@gmail.com; 4 JSC Research Institute of Cardiology and Internal Diseases, Almaty 050000, Kazakhstan; rustemtuleutayev@gmail.com; 5 Department of Internal Medicine, JSC Research Institute of Cardiology and Internal Diseases, Almaty 050000, Kazakhstan; kaibullayeva.j@gmail.com (J.K.); madinabaidildaeva707@gmail.com (M.B.); araims15@gmail.com (A.M.)

**Keywords:** chronic urticaria, disease activity, quality of life, urticaria control, UAS7, Kazakhstan, chronic spontaneous urticaria, chronic inducible urticaria, dermatology

## Abstract

**Background and Objectives**: Chronic spontaneous urticaria (CSU) significantly affects patients’ quality of life (QoL) and remains challenging to manage, particularly in under-researched regions. This study aimed to assess the clinical burden, disease control, and quality of life among patients with CSU in Kazakhstan and to identify predictors of severe disease activity. **Materials and Methods**: We conducted a cross-sectional study of 350 patients with CSU attending the Republican Allergy Center in Almaty between December 2024 and June 2025. Patients were classified based on the presence of co-existing chronic inducible urticaria (CIndU), angioedema, or both. Disease activity, control, and QoL were assessed using the Urticaria Activity Score over 7 days (UAS7), Urticaria Control Test (UCT), and Dermatology Life Quality Index (DLQI), respectively. Multivariable logistic regression and correlation analyses were used to identify predictors of severe disease and interrelationships among clinical measures. **Results**: Among 350 patients (mean age 43.5 ± 14.7 years; 78% female), 46.3% had CSU alone, while 53.7% had associated phenotypes. Severe urticaria (UAS7 ≥ 28) affected 30% of patients. Suboptimal disease control (UCT ≤ 11) was reported in 30%, and 30% experienced strong or very strong QoL impairment (DLQI > 10). Older disease onset (≥60 years; OR = 1.98; 95% CI: 1.02–3.81) and nighttime symptoms (OR = 1.67; 95% CI: 1.02–2.73) were independently associated with severe disease. A strong inverse correlation was observed between UAS7 and UCT (ρ = −0.71), and a positive correlation between UAS7 and DLQI (ρ = 0.66), highlighting the impact of disease activity on control and QoL. **Conclusions**: CSU imposes a substantial clinical and psychosocial burden in Kazakhstan. One-third of patients experience severe symptoms and impaired QoL despite ongoing treatment. Older age at disease onset and nighttime symptoms may serve as practical indicators of disease severity. These findings highlight the need for improved access to advanced therapies, systematic monitoring using validated tools, and multidisciplinary care strategies in resource-constrained settings.

## 1. Introduction

Chronic urticaria, defined as the presence of wheals, angioedema, or both lasting for six weeks or longer, is a prevalent and often debilitating dermatological condition [1]. Affecting approximately 1–2% of the global population at any time [1,2], chronic urticaria poses a significant public-health burden due to its persistent nature, unpredictable symptoms, and frequent underdiagnosis or mismanagement. Studies show that, on average, patients experience a diagnostic delay of about two years and consult more than six physicians before receiving a definitive diagnosis, underscoring inefficiencies in clinical recognition and referral pathways [3].

Despite its nonfatal nature, chronic urticaria can significantly impair patients’ quality of life during exacerbations, disrupting sleep, emotional well-being, and productivity, and placing a substantial burden on healthcare systems [3,4]. Studies indicate that anxiety is strongly associated with disease activity, suggesting a bidirectional relationship between psychological stress and urticaria severity [5].

Chronic urticaria typically resolves within two to five years; however, in about 30% of cases, symptoms may persist for a longer period [6]. Chronic urticaria is not only persistent but also often difficult to manage, as many patients do not respond to standard-dose antihistamines and require significantly higher doses for adequate symptom relief [7,8]. The current international treatment paradigm for chronic urticaria is stepwise and symptom-driven. Second-generation non-sedating antihistamines remain the first-line therapy, with dose escalation and the addition of omalizumab, cyclosporine, or other anti-inflammatory agents recommended for refractory cases [9,10]. Studies estimate that 40–60% of patients remain symptomatic despite treatment, and the actual proportion may be even higher in resource-constrainedsettings [3,11].

Chronic inducible urticaria (CIndU) and chronic spontaneous urticaria (CSU) can co-exist, with either condition potentially serving as the predominant urticaria phenotype, depending on clinical presentation [12,13]. Notably, CIndU is often associated with a longer disease duration than CSU, as suggested by recent studies [14]. CSU is the most common form of chronic urticaria, accounting for approximately two-thirds of all cases, and is frequently used as the reference phenotype in both clinical and epidemiological research [1,2,9].

Evaluating the proportion of patients who achieve disease control in real-world settings and identifying clinical predictors of uncontrolled or severe disease are crucial for improving patient outcomes. In Kazakhstan, the limited evidence on chronic urticaria—its disease control, activity and impact on disease-related quality of life—represents a significant knowledge gap. Furthermore, understanding the regional burden of chronic urticaria, including its phenotypic variability and impact on quality of life, is essential for informing healthcare policy and optimizing patient-care outcomes.

In light of these challenges, this study aimed to assess the clinical burden and quality of life among CSU patients in Kazakhstan. The study had two main objectives: (1) to identify clinical predictors of severe disease and (2) to evaluate the relationships between disease activity, treatment control, and the quality of life. By addressing an important evidence gap, the study aims to inform national strategies for the diagnosis, management, and long-term support of patients with CSU in Kazakhstan and comparable healthcare settings.

## 2. Materials and Methods

### 2.1. Study Design and Study Population

This cross-sectional study included 350 patients with CSU who were receiving treatment and attended outpatient visits at the Republican Allergy Center in Almaty, part of the Research Institute of Cardiology and Internal Diseases, between December 2024 and June 2025. The sample comprised a consecutive convenience sample of all eligible and consenting CSU patients presenting during the six-month recruitment period. Although no formal sample-size calculation was performed, this approach ensured inclusion of a representative real-world clinical population and provided sufficient statistical power for the planned multivariable analyses. The diagnosis of CSU was confirmed by reviewing medical records, from which additional clinical and demographic data were extracted, including age, sex, presence of angioedema, occurrence of nighttime symptoms, age at disease onset, and presence of coexisting CIndU. Based on this information, patients were categorized into one of the following chronic-urticaria phenotypes: chronic spontaneous urticaria (CSU), CSU with coexisting CIndU (CSU + CIndU), CSU with associated angioedema (CSU + Angioedema), and CSU with both CIndU and angioedema (CSU + CIndU + Angioedema). During their outpatient visits, all participants were informed about the study’s objectives and procedures. After providing written informed consent, participants completed three standardized questionnaires under physician supervision. The final sample size of 350 patients represented all eligible and consenting individuals within the defined recruitment period, ensuring a representative sample of the CSU patient population attending the clinic. The study protocol was approved by the Institutional Review Board of the Research Institute of Cardiology and Internal Diseases (approval number: 002-11-24, dated 27 November 2024).

### 2.2. Questionnaires Used

Three validated instruments were used to assess disease control, quality of life, and symptom activity among chronic urticaria patients.

Urticaria Control Test (UCT): The UCT assesses disease control for both spontaneous and inducible urticaria symptoms during the previous four weeks. It consists of four items, each scored from 0 to 4, yielding a total score ranging from 0 to 16. A score of ≥12 indicates well-controlled disease, whereas one of ≤ 11 suggests poor control. The internal consistency of the UCT in this study was excellent (Cronbach’s alpha = 0.89).

Dermatology Life Quality Index (DLQI): The DLQI evaluates the impact of dermatologic conditions on patients’ quality of life. It contains 10 items, with response options scored from 0 (“no impact”) to 3 (“very strong impact”), yielding a maximum score of 30. Total scores were interpreted as follows: 0–1 (no effect), 2–5 (mild), 6–10 (moderate), 11–20 (strong), and 21–30 (very strong impact). The DLQI also demonstrated high internal consistency in this population (Cronbach’s alpha = 0.91).

Urticaria Activity Score over 7 days (UAS7): The UAS7 measures the daily intensity of wheals and itching over a seven-day period. Each day’s score ranges from 0 to 6, resulting in a total weekly score of 0 to 42. Higher scores indicate greater disease severity.

### 2.3. Dependent and Independent Variables

The primary dependent variable in this study was disease activity, which was assessed using two analytical approaches. For the multivariable logistic regression, it was treated as a categorical variable, classified as mild-to-moderate or severe disease based on established UAS7 thresholds. Specifically, a UAS7 score of 0–27 indicated mild-to-moderate disease, whereas a score of 28 or higher was defined as severe disease activity. For the correlation analyses, urticaria activity was treated as a continuous variable, with the total UAS7 score used to assess relationships with other clinical and patient-reported outcomes.

The independent variables included in the multivariable logistic regression model were selected a priori based on clinical relevance and evidence from previous studies and international guidelines [1,12]. These predictors were chosen for their known or suspected associations with urticaria severity, control, or quality of life, and included age (continuous); sex (female as the reference group); age at disease onset (categorized as 1–44 years [reference], 45–59, and ≥60 years); presence of nighttime symptoms (yes/no); and CSU phenotype. The CSU phenotype, defined according to clinical presentation, included chronic spontaneous urticaria (CSU, reference category), CSU with concomitant CIndU, CSU with associated angioedema, and CSU with both CIndU and angioedema. The diagnosis of CIndU was made in accordance with national clinical protocols for urticaria in Kazakhstan and current international guidelines, based on patient history (anamnesis) and standardized provocation testing, when applicable [1,15].

### 2.4. Statistical Analysis Plan

Descriptive statistics were used to characterize the study population. Categorical variables were summarized as frequencies and percentages, while continuous variables were reported as means with standard deviations (SDs). Comparisons between chronic-urticaria phenotypes (CSU vs. other types) were conducted using Chi-square tests for categorical variables and Welch’s *t*-tests for continuous variables, the latter selected to account for potential variance inequality. To identify predictors of severe urticaria relative to mild or moderate disease, a multivariable logistic-regression model was constructed, adjusting for age and sex. Results were reported as adjusted odds ratios (AORs) with corresponding 95% confidence intervals (CIs). To explore associations among disease control, quality of life, and symptom burden, correlation analyses were performed using UCT, DLQI, and UAS7 scores. Both Spearman and Pearson correlation coefficients were calculated. Data visualizations included Spearman correlation heatmaps showing pairwise rho values and scatterplots combining density plots, point distributions, and correlation estimates. All statistical analyses were performed using R (version 4.3.1) and RStudio (version 2023.06.1+524) [16,17]. A two-tailed *p*-value of <0.05 was considered statistically significant. Section 2 provides sufficient detail to allow the replication and extension of the published results. All data included in the present analysis are available in Supplementary Table S1.

## 3. Results

Table 1 summarizes the demographic characteristics of the patients included in the study. Of the 350 patients analyzed, 162 (46.3%) were classified as having CSU alone, whereas 188 (53.7%) presented with other CSU phenotypes, including CSU with CIndU, angioedema, or both. The mean age of the study population was 43.5 ± 14.7 years. Patients with CSU were significantly older than those with other chronic urticaria phenotypes (46.8 ± 15.7 vs. 40.7 ± 13.3 years; *p* < 0.001). Overall, 78.0% of participants were female, with no statistically significant difference in sex distribution between CSU and other CSU phenotypes (*p* = 0.30). The mean UAS7 score for the total cohort was 19.1 ± 12.7, with no significant difference between CSU and other phenotypes (*p* = 0.80). Accordingly, severe urticaria (UAS7 ≥ 28) was observed in 30.0% of patients, affecting 32.7% of those with CSU and 27.7% of those with other phenotypes, with no statistically significant difference between the groups (*p* = 0.30). The mean UCT score was 11.7 ± 4.0, indicating that a substantial proportion of patients experienced suboptimal disease control. Overall, 70.0% of patients met the criterion for well-controlled disease (UCT ≥ 12), with comparable proportions among CSU patients (72.8%) and those with other CSU phenotypes (67.6%; *p* = 0.30). Quality-of-life impairment, assessed using the DLQI, was moderate at the population level, with a mean score of 8.6 ± 7.7. Patients with other CSU phenotypes reported higher DLQI scores than those with CSU alone (9.3 ± 7.8 vs. 7.7 ± 7.5), a difference that was marginally statistically significant (*p* = 0.051). Overall, 30.0% of patients reported a strong or very strong impact of chronic urticaria on quality of life, with no significant difference between groups (*p* = 0.30). Disease onset age also varied significantly by phenotype (*p* = 0.002). Early onset disease (1–44 years) was more frequent among patients with other chronic urticaria phenotypes (75.5%) compared with CSU patients (58.0%), whereas later onset (≥45 years) was more common in the CSU group. In contrast, the occurrence of nighttime symptoms did not differ significantly between CSU and other chronic urticaria phenotypes (31.5% vs. 26.1%; *p* = 0.30).

The results of the multivariable logistic regression analysis examining factors associated with severe urticaria are presented in Table 2. After adjustment for age and sex, disease onset at older age and the presence of nighttime symptoms were independently associated with increased odds of severe disease. Compared with patients whose disease onset occurred between 1 and 44 years, those with onset at 60–74 years had nearly a twofold higher likelihood of severe urticaria (OR = 1.98; 95% CI: 1.02–3.81; *p* = 0.042). In contrast, disease between 45 and 59 years was not significantly associated with disease severity (OR = 0.65; 95% CI: 0.33–1.20; *p* = 0.20). The presence of nighttime symptoms was also significantly associated with severe urticaria; patients reporting nocturnal symptoms had higher odds of severe disease (OR = 1.67; 95% CI: 1.02–2.73; *p* = 0.040) compared with those without such symptoms.

**Figure 1:**
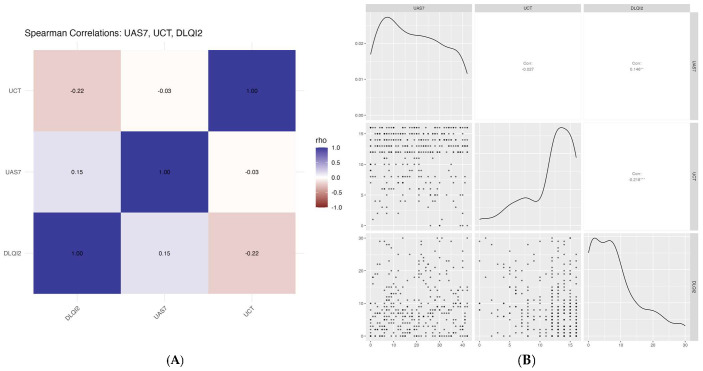
Correlations between urticaria activity, disease control, and quality of life among patients with CSU: (**A**) Spearman correlation heatmaps; (**B**) pairwise scatterplots. ** (*p*-value < 0.01); *** (*p*-value < 0.001).

Figure 1 illustrates the pairwise correlations between urticaria activity, disease control, and dermatology-related quality of life among patients with CSU. A strong inverse correlation was observed between UCT and UAS7, indicating that higher disease activity was consistently associated with poorer disease control. Similarly, UAS7 showed a positive correlation with DLQI, demonstrating that greater urticaria severity was correlated with greater quality-of-life impairment. In contrast, UCT was negatively correlated with DLQI, suggesting that better disease control is associated with lower quality-of-life impairment.

## 4. Discussion

This study provides novel insights into the clinical burden, disease control, and quality of life among patients with CSU in Kazakhstan. Of the 350 patients studied, approximately 80% were female, nearly one-third (30%) experienced severe disease activity as measured by the UAS7, and 30% reported a strong or very strong impairment in quality of life (DLQI > 10). Disease control, as measured by the UCT, was suboptimal in a considerable proportion of the cohort, with only 70% achieving well-controlled disease (UCT ≥ 12). Multivariable analysis identified older age at disease onset (≥60 years) and the presence of nighttime symptoms as independent predictors of severe CSU. These findings address the study’s primary aims: characterizing phenotype distribution, identifying risk factors for severe disease, and establishing correlations between disease activity, control, and quality of life. Together, these results highlight the multidimensional burden of CSU and provide critical epidemiological data from a previously underrepresented region.

Our findings align with international studies indicating that females experience a greater burden of CSU than males [18,19]. We also observed a significant association between older age at disease onset and increased disease severity, aligning with previous cohort studies suggesting that CSU in older adults may be more persistent and less responsive to standard therapies [20]. However, CSU in older individuals rarely occurs in isolation. Age-related immune dysregulation, comorbidities—including cardiovascular disease and thyroid dysfunction—and polypharmacy may complicate both diagnosis and management [2,20,21,22]. These factors may also confound disease severity assessments and treatment responses, underscoring the need for age-specific care pathways.

Our results also corroborate evidence indicating that approximately 30–50% of patients with CSU suffer from uncontrolled disease or significant quality-of-life impairment, even while receiving treatment [23]. Major challenges include refractory cases, coexisting conditions, the lack of curative therapies, and issues with omalizumab access or availability [23]. The strong inverse correlation between UAS7 and UCT scores observed in our study supports previous research demonstrating the sensitivity of these tools in assessing disease activity and control, as well as their impact on quality of life [24,25]. Notably, the high prevalence of comorbid CIndU (45.7%) in this cohort exceeds rates reported in previous studies, where it typically ranges from 27% to 43% [26,27,28,29,30]. This discrepancy may reflect regional differences in clinical phenotypes or variations in diagnostic practices [31]. Our study also reinforces prior evidence linking poor disease control with impaired quality of life and heightened psychological burden, including anxiety, depression, and sleep disturbances [32,33,34].

Taken together, our findings corroborate the hypotheses that CSU presents a complex, multidimensional burden. These results have several implications for clinical practice and healthcare planning in Kazakhstan and similar resource-constrained settings. The relatively high proportion of patients with severe symptoms and impaired quality of life underscores the need for earlier diagnosis, optimized treatment pathways, and improved access to third-line therapies, such as omalizumab, which may be underutilized due to cost or availability. Our results also support the routine integration of validated assessment tools—specifically the UAS7, UCT, and DLQI—to guide treatment decisions and monitor disease burden. Importantly, the identification of nighttime symptoms and older age at onset as predictors of severe disease may enable clinicians to stratify patients by risk and prioritize more aggressive management. Given the complex interplay between disease activity and psychological well-being, a multidisciplinary approach involving dermatologists, allergists, and mental health professionals may offer added benefit.

This study has several limitations. First, its cross-sectional design precludes causal inferences regarding the relationships between clinical variables, disease control, and quality of life. Second, the sample was drawn from a single tertiary referral center in Almaty, which may limit the generalizability of the findings to the broader Kazakhstani population or to patients managed in primary care settings. Third, the use of self-reported measures, including the DLQI and UCT, may be subject to recall and social desirability biases. However, the use of validated instruments with high internal consistency (Cronbach’s alpha > 0.89 for all scales) enhances the reliability of the results. Furthermore, while this study captured the distribution of CSU phenotypes within a national referral setting, chronic urticaria itself is a clinically heterogeneous condition. A more precise regional epidemiological characterization of CSU and CIndU subtypes, including phenotype overlap, would require pooled, multicenter datasets and prospective cohort designs. Such efforts would enable more robust phenotype validation and support the longitudinal assessment of disease progression across demographic strata, particularly in underrepresented and rural populations.

Another limitation of this study is that we were unable to assess the specific psychological burden or disease control profile associated with CSU with isolated angioedema. Future studies incorporating larger cohorts of patients with this specific phenotype are warranted to address this important clinical gap.

An additional limitation is the lack of assessment regarding the influence of other co-existing comorbidities, general quality-of-life domains beyond dermatology-specific impairment, and patient satisfaction with medical care. These outcomes are particularly relevant given the recent implementation of Kazakhstan’s Compulsory Social Health Insurance System (CSHIS), which has increased healthcare funding and broadened access to specialist services [35,36]. Evidence indicates that the CSHIS has had a significant impact on patient outcomes in Kazakhstan [37,38]. While these reforms potentially influence overall quality of life and treatment satisfaction among patients with CSU, such dimensions were not captured in the present study.

Importantly, our findings should be interpreted within the context of Kazakhstan’s healthcare system, which has made notable strides in expanding access to specialized care through the CSHIS. However, significant disparities persist between urban and rural populations, including unequal access to services, centralized procurement processes, and limited physician availability and clinical awareness in non-urban regions [39]. Notably, omalizumab is not currently reimbursed for chronic urticaria under the CSHIS or within the Guaranteed Volume of Free Medical Care, limiting access to this therapy for many patients [40]. Incorporating evidence-based CSU management into national dermatology and allergy guidelines, supported by ongoing clinician education and policy-level subsidies for advanced therapies, could substantially improve patient outcomes. In the broader context of global CSU management, our results reinforce the persistent gap between treatment guidelines and real-world clinical practice, particularly in low- and middle-income countries.

Future research should aim to (1) evaluate the longitudinal impact of CSU on mental health and occupational functioning; (2) examine the cost-effectiveness of early access to advance therapies within the national insurance model; and (3) explore the genetic or environmental contributors to phenotypic variation and treatment response. Prospective, multicenter cohort studies are particularly needed to guide personalized care strategies in Kazakhstan and similar healthcare settings.

## 5. Conclusions

In summary, the present study reveals that CSU imposes a substantial burden on patients in Kazakhstan, with one-third experiencing severe symptoms and a similar proportion reporting significant impairment in quality of life. Poor disease control remains prevalent, particularly among older individuals and those with nighttime symptoms. These findings are significant because they identify specific high-risk groups and highlight the need for improved clinical pathways and targeted interventions. We recommend expanding access to guideline-recommended therapies, integrating psychological support into routine care, and conducting longitudinal studies to track disease progression and treatment outcomes over time. Ultimately, a patient-centered and evidence-based approach is essential to mitigate the burden of CSU in Kazakhstan and other similar healthcare contexts.

## Figures and Tables

**Table 1:** Baseline characteristics of the study population by CSU phenotype.

Characteristic	CSU **N** = 162 ^1^	Other CSU Phenotype **N** = 188 ^1^	Overall **N** = 350 ^1^	*p*-Value ^2^
Age	46.75 (15.66)	40.72 (13.30)	43.51 (14.73)	<0.001
Gender				0.3
Female	130.0 (80.2%)	143.0 (76.1%)	273.0 (78.0%)	
Male	32.0 (19.8%)	45.0 (23.9%)	77.0 (22.0%)	
UAS7	19.29 (13.17)	18.98 (12.30)	19.12 (12.69)	0.8
UAS7 groups				0.3
Mild-to-moderate	109.0 (67.3%)	136.0 (72.3%)	245.0 (70.0%)	
Severe urticaria	53.0 (32.7%)	52.0 (27.7%)	105.0 (30.0%)	
UCT	11.90 (3.97)	11.59 (3.93)	11.73 (3.95)	0.5
UCT (well-controlled disease)	118.0 (72.8%)	127.0 (67.6%)	245.0 (70.0%)	0.3
DLQI2	7.69 (7.53)	9.30 (7.77)	8.55 (7.69)	0.051
DLQI groups				0.3
No, mild-to-moderate impact	118.0 (72.8%)	127.0 (67.6%)	245.0 (70.0%)	
Strong and very strong impact	44.0 (27.2%)	61.0 (32.4%)	105.0 (30.0%)	
Disease onset age group				0.002
1–44	94.0 (58.0%)	142.0 (75.5%)	236.0 (67.4%)	
45–59	43.0 (26.5%)	27.0 (14.4%)	70.0 (20.0%)	
60–74	25.0 (15.4%)	19.0 (10.1%)	44.0 (12.6%)	
Night symptoms				0.3
Present	51.0 (31.5%)	49.0 (26.1%)	100.0 (28.6%)	
Absent	111 (68.5%)	139 (74.9%)	250 (71.4%)	

^1^ n (%); Mean (SD); ^2^ Pearson’s chi-squared test; Welch’s two-sample *t*-test. Abbreviations: CIndU = chronic inducible urticaria; DLQI2 = Dermatology Life Quality Index; UAS7 = Urticaria Activity Score; UCT = Urticaria Control Test.

**Table 2:** Multivariable Logistic Regression of Factors Associated with Severe CSU.

Characteristic	N	OR	95% CI	*p*-Value
Disease onset age group	350			
1–44		—	—	
45–59		0.65	0.33, 1.20	0.2
60–74		1.98	1.02, 3.81	0.042
CSU phenotype	350			
CSU		—	—	
Other		0.79	0.50, 1.24	0.3
Night symptoms	350			
Absent		—	—	
Present		1.67	1.02, 2.73	0.040

Abbreviations: CI = confidence interval; CIndU = chronic inducible urticaria; OR = odds ratio.

## Data Availability

The original contributions presented in this study are included in this article. Further inquiries can be directed to the corresponding author.

## Supplementary Materials

The following supporting information can be downloaded at https://www.mdpi.com/article/10.3390/diagnostics16050672/s1: Table S1: Original data for patients with CSU in Kazakhstan.

## Abbreviations

The following abbreviations are used in this manuscript:

CI	Confidence Interval
CIndU	Chronic Inducible Urticaria
DLQI2	Dermatology Life Quality Index
OR	Odds Ratio
UAS7	Urticaria Activity Score
UCT	Urticaria Control Test
